# A Statistical Mechanics Approach to Describe Cell Reorientation Under Stretch

**DOI:** 10.1007/s11538-023-01161-4

**Published:** 2023-05-30

**Authors:** N. Loy, L. Preziosi

**Affiliations:** Politecnico di Torino, Torino, Italy

**Keywords:** Cell orientation, Fokker–Planck equations, Mechanotransduction, 74D05, 74L15, 92C10, 92C37, 35Q20, 35Q70, 35Q84

## Abstract

Experiments show that when a monolayer of cells cultured on an elastic substratum is subject to a cyclic stretch, cells tend to reorient either perpendicularly or at an oblique angle with respect to the main stretching direction. Due to stochastic effects, however, the distribution of angles achieved by the cells is broader and, experimentally, histograms over the interval $[0^\circ , 90^\circ ]$ are usually reported. Here we will determine the evolution and the stationary state of probability density functions describing the statistical distribution of the orientations of the cells using Fokker–Planck equations derived from microscopic rules for describing the reorientation process of the cell. As a first attempt, we shall use a stochastic differential equation related to a very general elastic energy that the cell tries to minimize and, we will show that the results of the time integration and of the stationary state of the related forward Fokker–Planck equation compare very well with experimental results obtained by different researchers. Then, in order to model more accurately the microscopic process of cell reorientation and to shed light on the mechanisms performed by cells that are subject to cyclic stretch, we consider discrete in time random processes that allow to recover Fokker–Planck equations through classical tools of kinetic theory. In particular, we shall introduce a model of reorientation as a function of the rotation angle as a result of an optimal control problem. Also in this latter case the results match very well with experiments.

## Introduction

**Fig. 1:**
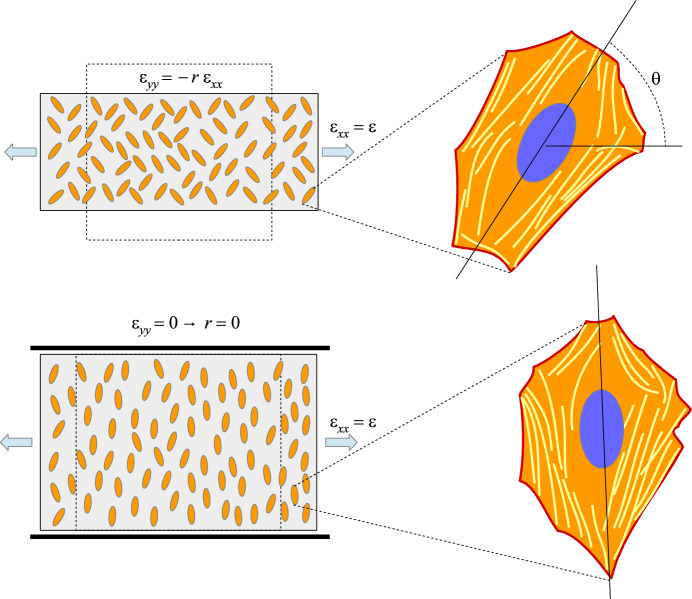
Experimental set-up and sketch of a sample oriented cell as schematized from actual pictures from Fig. 3a of Roshanzadeh et al. (2020). The top row refers to a case in which cells tend to orient at an oblique angle and the bottom row to the particular case in which ${\varepsilon }_{yy}=0$ (and then $r=0$) for which cells tend to orient perpendicularly to the main streching direction

In the 80’s the study of cardiovascular diseases led to the need of understanding the behaviour of cells of the heart and of the arterial walls subject to periodic deformations due to pulsatile heart contractions and consequent blood flow (Buck 1979, 1980). In order to mimick this environment, many authors seeded cells on a substratum that was stretched periodically (see, for instance, the recent review (Giverso et al. 2022) and references therein). It was generally found that for sufficiently high stretching frequencies (see Greiner et al. 2015; Hsu et al. 2009; Jungbauer et al. 2008; Lee et al. 2010; Tondon and Kaunas 2014) and amplitudes (see Boccafoschi et al. 2007; Dartsch et al. 1986; Kaunas et al. 2005; Mao et al. 2021; Morita et al. 2013), cells internally develop *stress fibers* that link to the substratum via *focal adhesions* and confer anisotropic characteristics to the cell (see Fig. 1). Such stress fibers are, at the equilibrium, mainly aligned perpendicularly to the main stretching direction or at oblique and symmetric angles with respect to it. Consequently, the cells take an elongated shape with the section of the nucleus that becomes elliptic with the long axis along the above directions as well. This fact well correlates with the observation that smooth muscle cells in the intima of arterial walls are oriented obliquely with respect to the vascular axial direction forming helical-like structures characterized by an angle with the longitudinal axis between $20^\circ$ and $40^\circ$ (Rhodin 1962; Shirinsky et al. 1989).

The reorientation dynamics in vitro is quite robust with respect to both cell type and experimental set-up. In fact, regarding the former aspect, fibroblasts, muscle-type cells, epithelial cells, endothelial cells, osteoblasts, melanocytes, mesenchymal stem cells, all respond in a similar way when periodically stretched. Regarding the latter aspect, the final result seems to be nearly independent from the applied frequency and amplitude, and from the mechanical characteristics of the substratum, with transitions when the corresponding values are smaller than some thresholds, i.e., too low frequencies, too small deformations, too soft substrata. On the other hand, the strain ratio in the two perpendicular directions turns out to be relevant, as well described by the experiments performed by Livne et al. (2014).

From the viewpoint of mathematical modelling, the first attempts to describe the phenomenon were based on a strain avoidance principle, consisting in the assumption that cells tend to reorient in the direction of minimal strain (Barron et al. 2007; Faust et al. 2011; Morioka et al. 2011; Wang 2000; Wang et al. 1995).

Successively, it was hypothesized that rather than minimal strain, the main reorientation direction tends to minimize stress (De et al. 2007, 2008; Livne et al. 2014). Therefore in these works, the evolution of the cell orientation $\theta$ is related to a linear elastic energy ${\mathcal {E}}$ through

1 $$\begin{aligned} \dfrac{d}{dt}\theta \propto -\dfrac{\partial }{\partial \theta }{\mathcal {E}}. \end{aligned}$$

In particular, Livne et al. (2014) modelled the ensemble of cells on the substratum as a linear elastic anisotropic material subject to biaxial strain and identified the equilibrium orientations $\theta _{eq}$ formed by the cell major axis or of the stress fibers and the direction of stretching corresponding to minimal energy. In this way, they found a linear relationship between $\cos ^2\theta _{eq}$ and a parameter quantifying the biaxiality of the deformation and the cell’s anisotropic material coefficients. They also showed that in this parameter plane, data obtained using fibroblasts tend to align along a straight line.

Starting from the observation that the experimental results holded true even for deformation ranges that make questionable the use of linear elasticity [they can go up to 30% (Faust et al. 2011; Livne et al. 2014)], Lucci and Preziosi (2021) proved that a generalization of the linear relationship found by Livne et al. (2014) also holds for a very large class of nonlinear constitutive orthotropic models. In the nonlinear framework, the squared cosine of the orientation angle is linearly dependent on a parameter which is the natural generalization of the one found by Livne et al. (2014), with a slope depending on a combination of elastic coefficients characterizing the nonlinear strain energy. A detailed bifurcation analysis is given. Also Lazopoulos and coworkers (Lazopoulos and Pirentis 2007; Lazopoulos and Stamenović 2006; Stamenović et al. 2009) employed a finite elasticity framework to describe stress fibers reorganization in strained cells, although they considered only uniaxial substratum stretching and addressed the problem using a non-convex energy, giving an explanation based on the co-existence of phases.

A viscoelastic model is instead proposed by Lucci et al. (2021) to explain why on the time scale of experiments the reorientation phenomenon does not occur for small frequencies, for instance, as a consequence of the reorganization of focal adhesions. A Maxwell-like force-deformation relation was also used by Chen et al. (2012) who focused on the dynamics of single stress fibers and on the reorganization of the attachment of focal adhesions to the substratum.

However, it must be noticed that for sake of simplicity most of the models mentioned above work in a deterministic framework, while, as in any biological process, randomness characterizes several aspects of the mentioned dynamics, such as the assembly and disassembly of stress fibers and of focal adhesions as well as the biochemical response inside the cell to such mechanical cues. Some of these aspects are considered in Hsu et al. (2009, 2010), Kaunas et al. (2011) where the focus is on the stochastic evolution of radially oriented stress fibers around the nucleus when the cell is subject to static and cyclic stretch. De (2018) focused instead on the stochastic stretch-sensitive bond association and dissociation processes taking also into account the elasticity of the cell-substratum system to predict the orientation and stability of adhesion mechanisms.

From the experimental point of view, the visible result of such uncertainties is reflected in a spread in cell orientation, in the sense that the distribution of the orientations of the cells is not represented by a Dirac delta, but by smoother functions. Actually, the outcome of the experiments is naturally described using histograms and graphs reporting the distribution of the frequencies of cell orientations falling in a partition of angle ranges over $[0^\circ ,~90^\circ ]$ (see, for instance, Barron et al. 2007; Chen et al. 2018; Faust et al. 2011; Hayakawa et al. 2001, 2000; Livne et al. 2014; Mao et al. 2021; Neidlinger-Wilke et al. 2001, 2002; Morioka et al. 2011; Wang et al. 1995; Wang and Grood 2000). The degree of spreading is not constant but depends on the amplitude and frequency of the imposed stretch. Specifically, it increases when decreasing amplitude and frequency.

The inclusion of some randomness allows the authors in Barron et al. (2007), Chen et al. (2018), Morioka et al. (2011), Wang et al. (1995) to compare the histograms obtained from the experiments with the curves obtained by the results of simulations of the orientation model that they propose. However, there, an analytical distribution function was not provided and the effect of stochasticity was not explored in detail.

One of the first analitycal treatments of the problem of describing the *probability density function* of the orientations of the cells (its time evolution or, at least, the stationary state) is provided by Kemkemer et al. (2006, 1999). They express the evolution of the orientation of a cell by means of an automatic controller, i.e. an ODE describing the temporal evolution of the single-cell orientation with an empirical forcing term that has the desired symmetry. They gain a stochastic differential equation (SDE) by adding diffusion, and obtain the evolution of the probability density function as a forward equation of the SDE. They can easily compute the stationary state of the resulting Fokker–Planck equation, represented by an exponential of a doubly-wrapped cosine, that is a Boltzmann-like distribution. In particular, they compare the analytical findings with experimental results and show that the Boltzmann-like distribution can describe cell orientations on curved substratums.

As a consequence, and as classically done in statistical mechanics when describing reorienting dipoles, many authors consider a Boltzman probability density function *f*

$$\begin{aligned} f(t,\theta ) \propto e^{-\dfrac{{\mathcal {E}}(\theta )}{kT}}, \end{aligned}$$

that is, as a matter of fact, coherent with the fact that the cells’ orientation evolves according to (1). Then, all the efforts lie in the modelling of the energy ${\mathcal {E}}$ of the system and of its temperature *T*. For example, starting from their already mentioned works (De et al. 2007, 2008), Safran and De (2009) describe the cell as a reorienting dipole subject to a periodic stretch and model the distribution of the orientations as a Boltzmann-distribution with a competition between the force determining the free energy of the dipole and the effective temperature. Faust et al. (2011) use this distribution assuming an ${{\mathcal {E}}}$ corresponding to the strain avoidance hypothesis. Also Mao et al. (2021) consider a Boltzmann-like distribution with an energy that is the sum of three contributions given by the work done by focal adhesions, by the pulling force, and by the elastic potential energy of bars in the tensegrity structure, that however presents a flaw. Driven by the aim of studying how peristalsic deformation affects the orientation of cells in the intestinal epithelial sheet, in a very recent work Gérémie et al. (2021), too, consider an SDE where the drift term is given by an elastic energy. They, then, determine the Fokker–Planck equation but they do not manage to retrieve a stationary probability density function, and they approximate it with a Gaussian distribution.

In the present work our aim is to determine the evolution and the stationary state of probability density functions describing the statistical distribution of the orientations of the cells subject to cyclic stretch. We shall do this using Fokker–Planck equations that we shall derive from microscopic stochastic processes taking into account the reorientation dynamics of cells in response to cyclic stretch. We shall then compare the probability density function with experimental results, in such a way that that the proposed microscopic process can be validated. As a first step, we shall consider a microscopic stochastic process ruled by the quadratic elastic energy proposed by Lucci and Preziosi (2021), that has already been investigated in a deterministic framework, leading to a good comparison with experimental results. As a second step, starting from the principle of minimization of the previous quadratic energy, we shall derive a microscopic stochastic process as a function of the actual rotation angles performed by the cell during the reorientation and caused by the cyclic stretch of the substratum. After deriving the probability density function describing the statistical distribution of the orientations of the cells that obey to this second stochastic process, we shall compare it with experimental results.

In order to do that, after recalling in Sect. 2 the mechanical background proposed by Lucci and Preziosi (2021), as a first step we shall model the evolution of the cell orientation by means of a stochastic differential equation in which the evolution of the direction is related to a general elastic energy plus a stochastic fluctuation (Sect. 3). In the same section the evolution of the probability density function is, then, classically obtained by means of a forward equation, namely a Fokker–Planck equation. We will find the stationary state and prove that it is an asymptotic equilibrium (Sect. 3.1). We will then show in Sect. 3.2 that using the elastic energy proposed by Lucci and Preziosi (2021) the results of the integration of the Fokker–Planck equation and its stationary state compare very well with the experimental results reported by Faust et al. (2011), Hayakawa et al. (2001), Jungbauer et al. (2008), Livne et al. (2014), Mao et al. (2021).

In Sect. 4, we shall describe the process of reorientation as a discrete in time stochastic process that happens with a certain frequency. After showing that the same Fokker–Planck equation used in Sect. 3 can be obtained by performing a *quasi-invariant limit* of the Boltzmann kinetic equation describing the evolution of the statistical distribution of the orientations of cells, in Sect. 4.2 we will propose a different viewpoint that consists in modelling reorientation as a result of an internal optimal control problem activated by the cell. Finally, we compare the results of the integration of the derived Fokker–Planck equation and of its stationary state, obtaining an even better agreement with respect to the one obtained in Sect. 3.2.

## Mechanical Backgrounds

We consider isolated cells that are seeded on the surface of a thin elastic substratum that is stretched biaxially. We define the *x*-axis along the direction subject to the maximum stretch. For sake of simplicity, we assume that cells behave elastically, are much softer than the substratum and strongly adhere to it, in such a way that the strain in the specimen is perfectly transferred to cells and is homogeneous. This translates in the fact that the strain tensor in the plane writes as ${\mathbb {E}}=\text {diag}({\varepsilon }_{xx},{\varepsilon }_{yy})=\text {diag}({\varepsilon },-r {\varepsilon })$ where *r* is called biaxiality ratio. As sketched in Fig. 1, when stretched, cells internally develop stress fibers that link to the substratum via focal adhesions. The fact that these stress fibers tend to form along a certain angle with respect to the stretch direction, confers anisotropic characteristics to the system. Neglecting substratum deformability by the traction forces exerted by the cells, of adhesion remodelling, and of viscoelastic effect in cell behaviour [that are however considered in a deterministic fashion in Lucci et al. (2021), Xu et al. (2016)], we will describe the system through a general orthotropic elastic energy denoted by ${\mathcal {U}}$ that will be affected by the cell orientations.

Referring to Fig. 1, we will denote by $\theta$ the cell orientation angle with respect to the *x*-axis. Now, at variance with what happens during migration when the moving cell polarizes forming a head and a tail, in this case the internal structures of the cell aligned along $\theta$ and along $\theta +\pi$ are geometrically indistinguishable [see, for instance, the work by Roshanzadeh et al. (2020), Tondon and Kaunas (2014), Wang et al. (1995)]. As a consequence, these angles are also equivalent from the energetic point of view, i.e. one must have ${\mathcal {U}}(\theta +\pi )={\mathcal {U}}(\theta )$. In addition, also the orientation of the axes is equivalent in the sense that it is observed experimentally that configurations $\theta$ and $\pi -\theta$ are equiprobable, as showed in Fig. 1. As a consequence, ${\mathcal {U}}(\pi -\theta )={\mathcal {U}}(\theta )$. Therefore, ${\mathcal {U}}(\theta )$ is an even $\pi$-periodic function and we can work under the following symmetry requirements

*U*1:       $${{\mathcal {U}}}(\theta )={{\mathcal {U}}}(2\pi -\theta )={{\mathcal {U}}}(\pi -\theta )={{\mathcal {U}}}(\pi +\theta ), \quad \forall \theta $$.

Most of the discussion that will follow is independent of the particular form of energy that is chosen provided that it possesses the symmetry properties above. However, using continuum mechanics arguments, it can be proved (see, for instance, the work by Ogden 2003) that an orthotropic elastic energy for a planar system with the symmetry properties in *U*1 depends on $\theta$ through the square of its cosine and is characterized by material coefficients describing the response to stretching along the orientation direction ($K_\Vert$) and perpendicular to it ($K_\perp$) and the response to shear ($K_s$), in addition to possible mixed terms.

To be specific in the following we will neglect mixed terms and use the following form of elastic energy

2 $$\begin{aligned}{} & {} {\mathcal {U}}=K_\Vert \varepsilon ^2 \bar{{\mathcal {U}}}=\dfrac{K_\Vert \varepsilon ^2}{2} \left\{ \left[ (r+1)\cos 2\theta +1-r\right] ^2\right. \nonumber \\{} & {} \left. \qquad +{\tilde{K}}_{\bot }\left[ (r+1)\cos 2\theta -1+r\right] ^2+{\tilde{K}}_s(r+1)^2 (1-\cos ^2 2\theta )\right\} , \end{aligned}$$

where ${\tilde{K}}_{\bot }=\dfrac{K_{\bot }}{K_{\Vert }}$ and ${\tilde{K}}_s=\dfrac{K_s}{K_{\Vert }}$. We notice that Eq. (2) is a generalization of the energy used by Livne et al. (2014) and a particular case of the one used by Lucci and Preziosi (2021), Lucci et al. (2021). Of course, it satisfies the symmetry requirements *U*1.

Referring then to Lucci and Preziosi (2021), Lucci et al. (2021) for a more detailed stability analysis, defining

$$\begin{aligned} \rho (\alpha )=\frac{1+\alpha }{1-\alpha }=\frac{2-{{\tilde{K}}}_s}{\tilde{K}_s-2{{\tilde{K}}}_\bot }, \end{aligned}$$

the following scenarios are possible in terms of *r* and

3 $$\begin{aligned} \alpha= & {} \dfrac{1+{\tilde{K}}_{\bot }-{\tilde{K}}_s}{1- {\tilde{K}}_{\bot }}: \end{aligned}$$

**Fig. 2:**
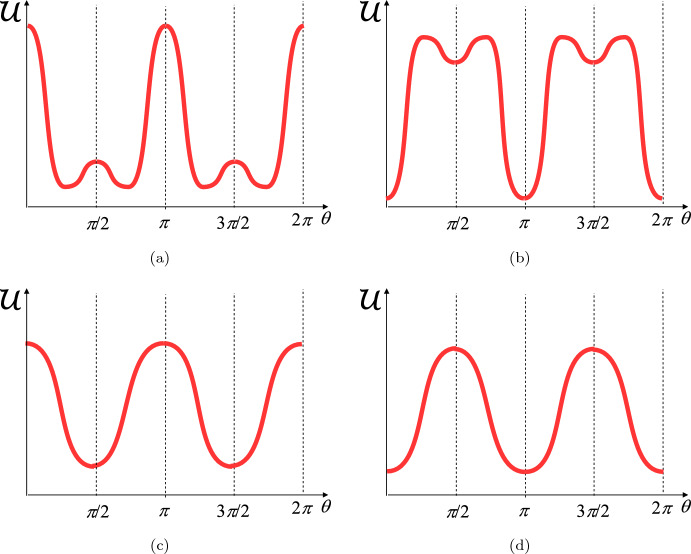
Elastic energy scenarios: **a** corresponds to Case 1, **b** to Case 2, **c** to Case 3 and **d** to Case 4

**Case 1:** $\forall r$ if $\alpha >1$ and for $r\in \left[ \frac{1}{\rho (\alpha )},\rho (\alpha )\right]$ if $\alpha \in (0,1)$, there is only one stable equilibrium $\theta _{eq}\in \left( 0,\frac{\pi }{2}\right)$ such that
  4 $$\begin{aligned} \cos ^2\theta _{eq}=\dfrac{1}{2}+\dfrac{1}{\alpha }\left( \dfrac{1}{2}-\dfrac{1}{r+1}\right) , \end{aligned}$$
  or
  5 $$\begin{aligned} \cos 2\theta _{eq}=\dfrac{1}{\alpha }\,\dfrac{r-1}{r+1}. \end{aligned}$$
  Therefore, due to *U*1, there are four stable equilibria in $[0,2\pi )$, namely in $\theta _{eq}^1=\theta _{eq}$, $\theta _{eq}^2=\pi -\theta _{eq}$, $\theta _{eq}^3=\pi +\theta _{eq}$, $\theta _{eq}^4=2\pi -\theta _{eq}$ (see Fig. 2a).
**Case 2:** $\forall r$ if $\alpha <-1$ and for $r\in \left[ \rho (\alpha ),\frac{1}{\rho (\alpha )},\right]$ if $\alpha \in (-1,0)$, there are four stable equilibria in $[0,2\pi )$, namely $\theta _{eq}^1=0$, $\theta _{eq}^2=\pi /2$, $\theta _{eq}^3=\pi$, $\theta _{eq}^4=3\pi /2$ (see Fig. 2b);
**Case 3:** for $r<\rho (\alpha )$ if $\alpha \in (-1,0)$ and $r<\frac{1}{\rho (\alpha )}$ if $\alpha \in (0,1)$, there are two stable equilibria in $[0,2\pi )$, namely $\theta _{eq}^1=\theta _{eq}^2=\pi /2$, $\theta _{eq}^3=\theta _{eq}^4=3\pi /2$ (see Fig. 2c);
**Case 4:** for $r>\frac{1}{\rho (\alpha )}$ if $\alpha \in (-1,0)$ and $r>\rho (\alpha )$ if $\alpha \in (0,1)$, there are two stable equilibria in $[0,2\pi )$, namely $\theta _{eq}^1=\theta _{eq}^4=0$, $\theta _{eq}^2=\theta _{eq}^3=\pi$ (see Fig. 2d).

We remark that in experimental works, observations of the orientations are reported over $[0,\pi /2)$ when the parameters of the experimental setting correspond to cases (a) and (b), while the observations are reported over $[0,\pi )$ when the parameters of the experimental setting correspond to cases (c) and (d). In particular, in the following we shall be interested in experimental settings that lead to scenarios (a) and (c) (Case 1 and 3). We want to highlight the fact that when dealing with scenario (a), experimentalists tend to represent data over $[0,\pi /2)$ as, because of the aforementioned symmetry around the axis, the measure of $\theta \in [\pi /2,\pi )$ is reported in the histogram bin corresponding to $\pi -\theta \in [0,\pi /2)$.

Working in a deterministic framework, on the basis of Lagrangian mechanics arguments, we can relate the evolution in time of the orientation angle $\theta$ with the changes in the virtual work ${\mathcal {L}}$ done by the stress acting on the cell due to stress fiber alignment. Considering an overdamped regime, which corresponds to neglecting inertial effects, we can then write

6 $$\begin{aligned} 0=-\eta \dfrac{d\theta }{dt}-\dfrac{\partial {\mathcal L}}{\partial \theta }\,, \end{aligned}$$

where $\eta$ is a viscous-like coefficient measuring cell resistance to internal rearrangement of stress fibers. In the elastic case Eq. (6) reduces to

7 $$\begin{aligned} \eta \dfrac{d\theta }{dt}=-\dfrac{\partial {\mathcal {U}}}{\partial \theta }(\theta ,t)\,, \end{aligned}$$

or

8 $$\begin{aligned} \dfrac{d\theta }{dt}=-\,\dfrac{\varepsilon ^2(t)}{\lambda _\theta }\,\dfrac{\partial \bar{{\mathcal {U}}}}{\partial \theta }(\theta )\,, \end{aligned}$$

where $\lambda _\theta =\eta /K_\Vert$ and we have put in evidence that the strain might be time-dependent.

Referring to the work by Lucci et al. (2021) for a more detailed discussion, we here observe that the same equation is obtained for a viscoelastic Maxwell-like model in the limit of high frequencies $\omega$ with respect to the inverse of the viscoelastic relaxation time $\lambda$, i.e., $\lambda \omega \gg 1$. On the contrary, in the limit $\lambda \omega \ll 1$ viscous effects dominate and a term $\lambda \omega$ appears at the numerator (related to the appearance of a strain rate, i.e., ${\varepsilon }(t){\dot{{\varepsilon }}}(t)$ instead of ${\varepsilon }^2(t)$), so that the effective $\lambda _\theta$ becomes $\frac{\lambda _\theta }{\lambda \omega }$. Considering that $\lambda$ is of the order of one minute for both stress fiber and focal adhesion remodelling (Chen et al. 2013; Pasapera et al. 2010), one has that the transition from low to high frequencies occurs for $\omega$ about 0.01–0.1 Hz. In the work by Lucci and Preziosi (2021), Lucci et al. (2021) the authors perform simulations of the deterministic process (8), showing a very good agreement with experimental results that report the average orientation of cells subject to cyclic stretch.

At variance with the previous deterministic description and, as any biological process, cell reorientation is strongly affected by their stochastic behaviours. From the experimental point of view, then, this leads to a representation of the orientation state of the ensemble of cells in terms of mean, variance, and, whenever possible, frequency histograms, as discussed in the following (see Figs. 5, 6 and 7). In parallel, from the theoretical point of view, this leads to the need of determining a probability density function describing the statistical distribution of the orientations. For this reason, in the following, we will introduce a statistical mechanics approach.

## Statistical Description of the Orientations of Cells under Bi-axial Stretch

In order to describe analytically the statistical distribution of the orientations of the cell, we introduce the probability density function $f=f(t,\theta )$ such that $f(t,\theta )d\theta$ is the fraction of cells having orientation in $[\theta , \theta +d\theta ]$ at time *t*. As discussed above, the fact that cells have no identifiable head and tail, implies that if a cell is rotated by $\pi$, it is not possible to perceive a difference in cell orientation. Hence the angles $\theta$ and $\theta +\pi$ identify the same orientation. Therefore we shall deal with $\pi$-periodic probability density functions *f*, so that $f(t,\theta )=f(t,\theta +k\pi ) \, \forall k \in {\mathbb {Z}}$. In addition, as a probability density function, *f* must satisfy

*F*1:       $$f \ge 0$$;
*F*2:       $$\displaystyle {\int _{0}^{\pi }} f(t,\theta ) \, d\theta =1$$.

Moreover, due to the symmetry related to the choice of the direction of the axes along the principal strain directions, the following property also holds

*F*3:       $$f(t,0)=f(t,\pi ), \, \forall t\ge 0$$;
*F*4:       *f* satisfies the same symmetry property as *U*1, i.e. $f(t,\theta )$ is s.t. $f(t,\pi -\theta )=f(t,\theta )$;

where *F*3 is also implied by the periodic character of the distribution function *f*.

With the aim of taking randomness into account, we may add a stochastic fluctuation to (7),

9 $$\begin{aligned} \dfrac{d\theta }{dt}=-\dfrac{1}{\eta }\dfrac{\partial {{{\mathcal {U}}}}}{\partial \theta } + \sqrt{\dfrac{\sigma ^2}{\lambda _\theta }}\xi \,, \end{aligned}$$

where $\xi$ is a Gaussian random variable with zero mean and unitary variance and $\sigma$ takes into account the stochastic fluctuations due to uncertainties. The latter may then be more properly rewritten as an It$\hat{\text {o}}$ process

10 $$\begin{aligned} d\theta =-\dfrac{1}{\eta }\dfrac{\partial {{{\mathcal {U}}}}}{\partial \theta }dt +\sqrt{\dfrac{\sigma ^2}{\lambda _\theta }}dW_t\,, \end{aligned}$$

where $dW_t=\sqrt{t}\xi$, being, then, $W_t$ a Wiener process.

The Fokker–Planck equation describing the forward evolution of the probability density distribution *f* of the orientations of the cells that follow the dynamics (10) is then (Risken 1996)

11 $$\begin{aligned} \dfrac{\partial }{\partial t}f(t,\theta )=\dfrac{{\varepsilon }^2(t)}{\lambda _\theta }\,\dfrac{\partial }{\partial \theta } \left( \dfrac{\partial \bar{{\mathcal {U}}}}{\partial \theta }(\theta )f(t,\theta )\right) + \dfrac{1}{2\lambda _\theta }\dfrac{\partial ^2 }{\partial \theta ^2}\left( {\sigma ^2} f(\theta ,t)\right) . \end{aligned}$$

We observe that though in most experiments ${\varepsilon }(t)={\varepsilon }(1-\cos \omega t )$, since we are interested in modelling the process of cell reorientation, as it is classically done in previously discussed elastic models, we will consider the mean strain ${\varepsilon }$ over an oscillation period.

Introducing the nondimensional time ${\bar{t}}=\dfrac{t {\varepsilon }^2}{\lambda _\theta }$, then the Fokker–Planck equation describing the evolution of ${\bar{f}}({\bar{t}},\theta )=f({\bar{t}}\lambda _{\theta }/{\varepsilon }^2,\theta )$ reads

12 $$\begin{aligned} \dfrac{\partial }{\partial {\bar{t}}}{\bar{f}}({\bar{t}},\theta )= \dfrac{\partial }{\partial \theta }\left( \dfrac{\partial \bar{{\mathcal {U}}}}{\partial \theta }{\bar{f}}({\bar{t}},\theta )\right) + \dfrac{\partial ^2 }{\partial \theta ^2}\left( {\bar{\sigma }}^2 {\bar{f}}({\bar{t}},\theta )\right) \end{aligned}$$

where ${\bar{\sigma }}^2=\dfrac{\sigma ^2}{2{\varepsilon }^2}$. This already puts in evidence that increasing the stretch amplitude decreases the dimensionless diffusion coefficient ${\bar{\sigma }}$ leading to a more focused response and more peaked distribution functions, and vice versa.

As already recalled, the inclusion of viscoelastic effects leads to the same results in the high frequency regime. On the other hand, in the low frequency regime, the dimensional analysis is modified because ${\varepsilon }^2$ is formally replaced by ${\varepsilon }^2\lambda \omega$. As a consequence, the effective dimensionless diffusion coefficient is ${\bar{\sigma }}^2=\dfrac{\sigma ^2}{2\lambda \omega {\varepsilon }^2}$, showing that when the imposed frequency decreases, $\bar{\sigma }$ increases leading to broader probability density functions.

We remark that Eq. (12) is similar to the one analyzed by Bastardis et al. (2008), and by Coffey et al. (2009) where the authors study a Fokker–Planck equation with a periodic potential that rules the rotational motion of a Brownian particle with inertial effects and that has the same symmetry properties as the elastic energy (2). In particular they extend Kramer’s escape theory (Bastardis et al. (2008)) and treat a similar problem in the context of superparamagnetic relaxation of magnetic nanoparticles in 3D in Coffey et al. (2009).

### The Stationary Equilibrium

Dropping the bars over *f* and *t* here and henceforth, if we denote by

13 $$\begin{aligned} {\mathcal {F}}[\theta ,f(t,\theta )]= \dfrac{\partial \bar{{\mathcal {U}}}}{\partial \theta }(\theta ) f(t,\theta ) +\dfrac{\partial }{\partial \theta }\left( {\bar{\sigma }}^2 f(t,\theta )\right) , \end{aligned}$$

then the $\pi$-periodicity of $\bar{{\mathcal {U}}}$ and *f*, implies that

14 $$\begin{aligned} {\mathcal {F}}[\pi ,f(t,\pi )]={\mathcal {F}}[0,f(t,0)]. \end{aligned}$$

In particular, thanks to the differentiability of $\bar{{\mathcal {U}}}$, the stationary solution $f^{\infty }$ of (12), coupled with an initial condition $f_0$, satisfying *F*1, *F*2, *F*3 is found by imposing

15 $$\begin{aligned} {\mathcal {F}}[\theta ,f^{\infty }(\theta )]=0, \end{aligned}$$

where the r.h.s. side is zero because of the boundary conditions (14). Thus, the stationary state of (12) is

16 $$\begin{aligned} f^{\infty }(\theta )=C\exp \left( -\dfrac{\bar{{\mathcal {U}}}(\theta )}{{\bar{\sigma }}^2} \right) , \end{aligned}$$

where *C* is a normalization constant. We observe that the maxima (resp. minima) of $f^{\infty }(\theta )$ correspond to minima (resp. maxima) of $\bar{{\mathcal {U}}}$. In particular, recalling that *f* is defined in $[0,\pi )$, in Cases 3 and 4 there is only a maximum respectively in $\frac{\pi }{2}$ and 0. Therefore, in the former case, due to symmetry, the mean corresponds to the mode. A similar property can be obtained in the latter case working in the more convenient periodicity interval $\left( -\,\frac{\pi }{2},\frac{\pi }{2}\right]$, otherwise the mean is trivially and misleadingly equal to $\frac{\pi }{2}$. On the other hand, in Cases 1 and 2, $f^{\infty }(\theta )$ is a bi-modal distribution in $[0,\pi )$ with modes $\theta _{eq}^1,\pi -\theta _{eq}^1$ and 0, $\frac{\pi }{2}$, respectively. Actually, for the already mentioned symmetry reasons, usually, the range of angles used to report experimental data is the first quadrant $[0,\pi /2)$, rather than $[0,\pi )$ or $[0,2\pi )$. In this case, then, the notion of mean looses its informative role, especially with respect to the mode that, restricted to $[0,\pi /2)$ is $\theta _{eq}^1$ in Case 1.

#### Remark 1

We observe that, if $\sigma =0$, i.e. there is no stochastic fluctuation in (10), then the stationary state given by imposing (15) is a Dirac delta or a weighted sum of Dirac deltas centered in the stable equilibria orientations.

As usually done for the standard Fokker–Planck equation (Furioli et al. 2017), convergence to the stationary state can be studied by analyzing the monotonicity in time of various Lyapunov functionals of the solution. The typical one is the relative Shannon entropy

17 $$\begin{aligned} H(f,f^{\infty })=\int _{0}^{\pi }f(\theta ,t) \log \left( \dfrac{f(t,\theta )}{f^{\infty }(t,\theta )}\right) \, d\theta \,, \end{aligned}$$

where $f,f^{\infty }: I \subset {\mathbb {R}}\rightarrow {\mathbb {R}}_+$ aree two probability densities. As periodic boundary conditions (14) hold, it is straightforward to prove (see Furioli et al. 2017) that the Shannon entropy monotonically decreases in time towards the stationary state, i.e.

$$\begin{aligned} \dfrac{d}{dt}H(f,f^{\infty }) \le 0\quad \text {and} \quad \dfrac{d}{dt}H(f,f^{\infty }) = 0 \quad \text {iff} \quad f=f^{\infty }. \end{aligned}$$

Therefore, $f^{\infty }$ is an asymptotic global equilibrium state.

### Statistical Description and Comparison with Experiments

Usually, dealing with angles requires circular statistics and the definition of trigonometric moments (Mardia and Jupp 1999), e.g. the circular mean

$$\begin{aligned} \langle \theta (t)\rangle :=\arctan \dfrac{\beta (t)}{\alpha (t)}, \quad \alpha = \int _0^{\pi }\cos \theta f(t,\theta ) \, d\theta , \quad \beta = \int _0^{\pi }\sin \theta f(t,\theta ) \, d\theta . \end{aligned}$$

However, the symmetry properties of *f* in Case a) and Case b), that prescribe a bi-modal probability density function, would always lead to $\alpha =0$ and, therefore, $\langle \theta (t)\rangle =\frac{\pi }{2}$. For this reason, we will use the following definition restricted to the first quadrant

18 $$\begin{aligned} \bar{\theta }_c(t):=\arctan \dfrac{\displaystyle \int _0^{\pi /2}\sin \theta f(t,\theta ) \, d\theta }{\displaystyle \int _0^{\pi /2}\cos \theta f(t,\theta ) \, d\theta }, \end{aligned}$$

even because it better correlates with the definition of average

19 $$\begin{aligned} \bar{\theta }_\ell (t):=2\displaystyle \int _0^{\pi /2} \theta f(t,\theta ) \, d\theta , \end{aligned}$$

that has been used in most experimental papers, where the 2 accounts for renormalization over $[0,\pi /2)$. We will also use the coherent definition of variance

20 $$\begin{aligned} {\bar{v}}_\ell (t):=2\displaystyle \int _0^{\pi /2} (\theta -\bar{\theta }_\ell )^2 f(t,\theta ) \, d\theta . \end{aligned}$$

An index $\infty$ will identify the quantities above computed for the equilibrium distribution $f^{\infty }$.

**Fig. 3:**
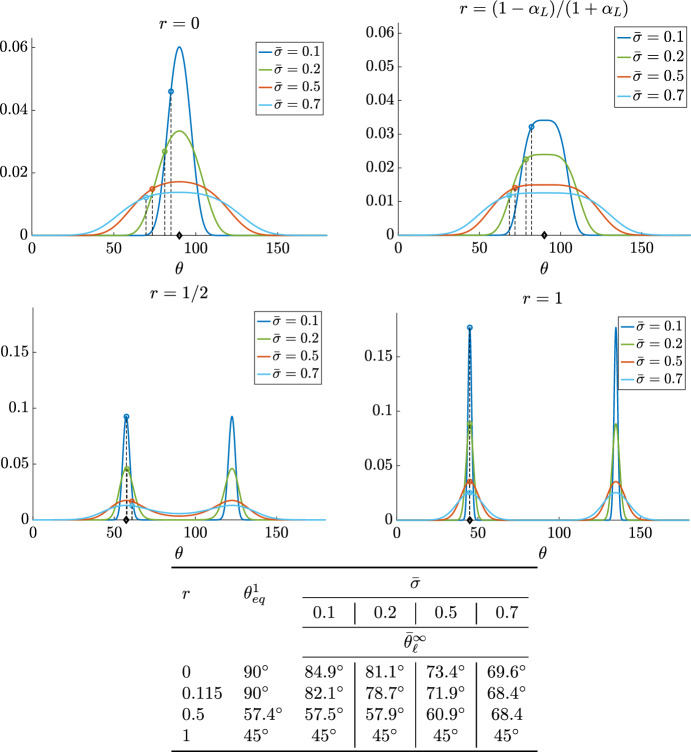
Profile of the stationary state (16) with $\bar{{\mathcal {U}}}$ given by (2) for various values of ${\bar{\sigma }}$ and *r* as specified in the title and legend of the figures. In all figures ${\tilde{K}}_{s}=0.7$. The value $r=\rho (\alpha _L)=\frac{1-\alpha _L}{1+\alpha _L}=0.115$ refers to the bifurcation point. The table reports $\theta _{eq}^1$ (denoted by a $\varvec{\diamond }$ in the figures) obtained by (4) and the mean $\bar{\theta }_{\ell }^{\infty }$ over $\left[ 0,\frac{\pi }{2}\right)$ (denoted by a circle in the figures), computed using (19) with $f^{\infty }$ defined by (16)

**Fig. 4:**
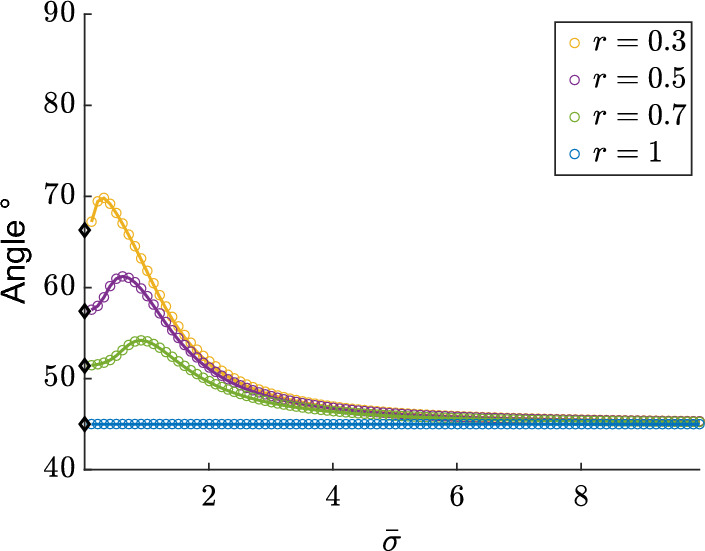
Average orientation as a function of ${\bar{\sigma }}$ and for different values of *r*. $\bar{\theta }_c^{\infty }$ (circles) and $\bar{\theta _\ell }^{\infty }$ (full line) are computed, respectively, using (18), (19) and (16). Linear and circular average coincide. Moreover, increasing values of ${\bar{\sigma }}$ lead to $\pi /4$, corresponding to uniform distributions, while for small values of ${\bar{\sigma }}$ the average tends to $\theta _{eq}^1$ (marked by $\varvec{\diamond }$).

However, some remarks are needed. First of all, we observe that in general the average and the mode do not coincide, i.e. $\bar{\theta }^{\infty }_c, \bar{\theta }^{\infty }_\ell \ne \theta _{eq}^1$. They obviously do when $\sigma \rightarrow 0$. However, we will see numerically (see Fig. 4) that in most cases $\bar{\theta }_c^{\infty }=\bar{\theta }^{\infty }_\ell$. In order to clarify this point, in Fig. 3 we plot the equilibrium distribution (16) over the interval $[0,\pi )$ for different values of the parameters *r* and ${\tilde{K}}_s$, $\alpha$ being fixed to the value $\alpha _L=0.794$ determined fitting the data of the experiments by Livne et al. (2014). Then we vary ${\tilde{K}}_s$ and, from (3), set

21 $$\begin{aligned} {\tilde{K}}_{\bot }=\dfrac{{\tilde{K}}_s-1+\alpha _L}{1+\alpha _L}. \end{aligned}$$

The positivity of ${\tilde{K}}_{\bot }$ prescribes the compatibility condition

22 $$\begin{aligned} {\tilde{K}}_s>1-\alpha _L. \end{aligned}$$

Therefore, we remark here that for all the figures presented most of the parameters are imposed by the experimental setting ($r, \alpha =\alpha _L$, ${\tilde{K}}_{\Vert }$, ${\tilde{K}}_{\bot }$, ${\tilde{K}}_{s}$). The values of ${\tilde{K}}$ are not in general given by experimentalists, but ${\tilde{K}}_{\Vert }=1$, ${\tilde{K}}_{\bot }$ is given by (21), and the only free parameters are ${\tilde{K}}_s$, that must obey constraint (22), and $\sigma$. In particular we have observed that ${\tilde{K}}_s$ does not influence the average of the distribution, as well as its shape, and we shall always consider ${\tilde{K}}_s=0.7$, while the greatest role is played by $\sigma$.

We remark that in Fig. 3 and in all the other Figures, we have preferred to describe angles in degrees rather than in radians for a better readability and a more direct comparison with the statistical descriptions of the experimental results.

In addition to the obvious observation that the diffusion parameter ${\bar{\sigma }}$ influences the spread of the orientations, other two facts linked to the presence of the diffusion stochastic term emerge explicitly and are put in evidence in Fig. 4:

- unless for the symmetric case $\theta _{eq}^1=\frac{\pi }{4}$ that is always obtained for $r=1$ (see Eq.(4)), the average of the probability density distribution computed over $\left[ 0,\frac{\pi }{2}\right)$ does not correspond to $\theta _{eq}^1$, that is identified by the mode in the first quadrant, i.e. the maximum of the distribution function;
- the average of the probability density depends on $\sigma$ and tends to the mode $\theta _{eq}^1$ (marked by ${\varvec{\diamond }}$) when $\sigma \rightarrow 0$ and to $\pi /4$, corresponding to a uniform distribution, when $\sigma \rightarrow +\infty$.

In Fig. 4 we also observe that the linear and the circular average at the stationary state coincide. Therefore, as experiments always consider the linear average, then in the following we shall make reference to $\bar{\theta }_\ell$. We remark that, for values of the average that are close to $\theta _{eq}$, there may be two different values of $\sigma$ and therefore two different probability density functions that allow to recover the same average $\bar{\theta }_\ell$ (see Fig. 4). Therefore, at each time we shall determine the one that better reproduces experimental results. It is evident that in Case 3 when $\theta ^1_{eq}=\theta ^2_{eq}=\frac{\pi }{2}$, then it is more appropriate to use $\langle \theta \rangle$, rather than ${\bar{\theta }}_\ell$.

**Fig. 5:**
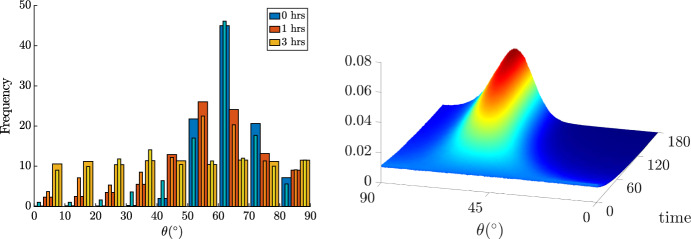
Comparison of the evolution of the probability density function obtained by performing a Monte Carlo simulation of (10) with the experimental data reported in Hayakawa et al. (2001). In particular, $\varepsilon =20\%$ and $r=0.4$. Solution for $\theta _{eq}^1\approx 61^\circ$, $\sigma \approx 0.04$, and $\lambda _\theta \approx 0.18$ s. On the left, the thick bars in blue, red and yellow refer to the simulation results at $t=0,1,3$ hours, respectively, while the corresponding lighter and thinner bars correspond to experimental datas. On the right, evolution of the probability density function.

With the aim of comparing the probability density functions with experimental results, we now focus on some papers reporting histograms of the percentage of cells in intervals of orientation angles. As in most cases esperimental data are given for $\theta \in \left[ 0,\frac{\pi }{2}\right]$, we will restrict to the first quadrant.

In Fig. 5 we compare the temporal evolution of the probability density distribution obtained by integrating (10) with a Monte Carlo approach with the experimental data reported in the work by Hayakawa et al. (2001) (the represented histograms) for $\varepsilon =20\%$, $r=0.4$ and $\omega =1$ Hz, that implies that we are in a high frequency regime. In these experiments it is found that at $t=1$ h the average orientation is $52.8^{\circ }$, while at $t=3$ hours, when more than the $80\%$ of the cells are oriented at angles of $50^{\circ }$–$80^{\circ }$, the average orientation is $62.02^{\circ }$. Using (4) and $\alpha =\alpha _L$ the minimum of the elastic energy is obtained at $\theta _{eq}^1\approx 61^\circ$. In particular, we have run a Monte Carlo simulation of (10) with $N=10^6$ cells and $dt=0.06$ s, the initial probability density function is the uniform distribution over $[0,\pi )$. We have recovered the probability density function as an histogram of the orientations of the simulated particles that, thus, approximates the solution to (12). In particular, we remark that the simulation is run over $[0,\pi )$. We then restrict and renormalize *f* over $[0,\pi /2)$ for comparison purposes with the reported histograms. Then, we calibrated $\sigma$ in order to obtain a stationary state with average $62.2^{\circ }$ and that is closer to the histograms presented in Fig. 5 (left panel) and $\lambda _\theta$ to replicate the time evolution of data. In particular, we set $\sigma \approx 0.04$ that is such that $\bar{\theta }_\ell =62.2^{\circ }$ and the probability density function has the same height as the histogram at $t=3$  h and $\lambda _\theta \approx 0.18$  s. After 1 hour we have that the average orientation is $54.6^{\circ }$ and after 3 hours the average orientation is $62.04^{\circ }$ and $85\%$ of the cells is oriented at angles of $50^{\circ }$–$80^{\circ }$. In Fig. 5 (left panel) we plot both the histograms with classes’ width of 10 degrees and, in the righ panel, the time evolution of the recovered probability density functions (that are histograms with classes width of 0.01 degrees).

**Fig. 6:**
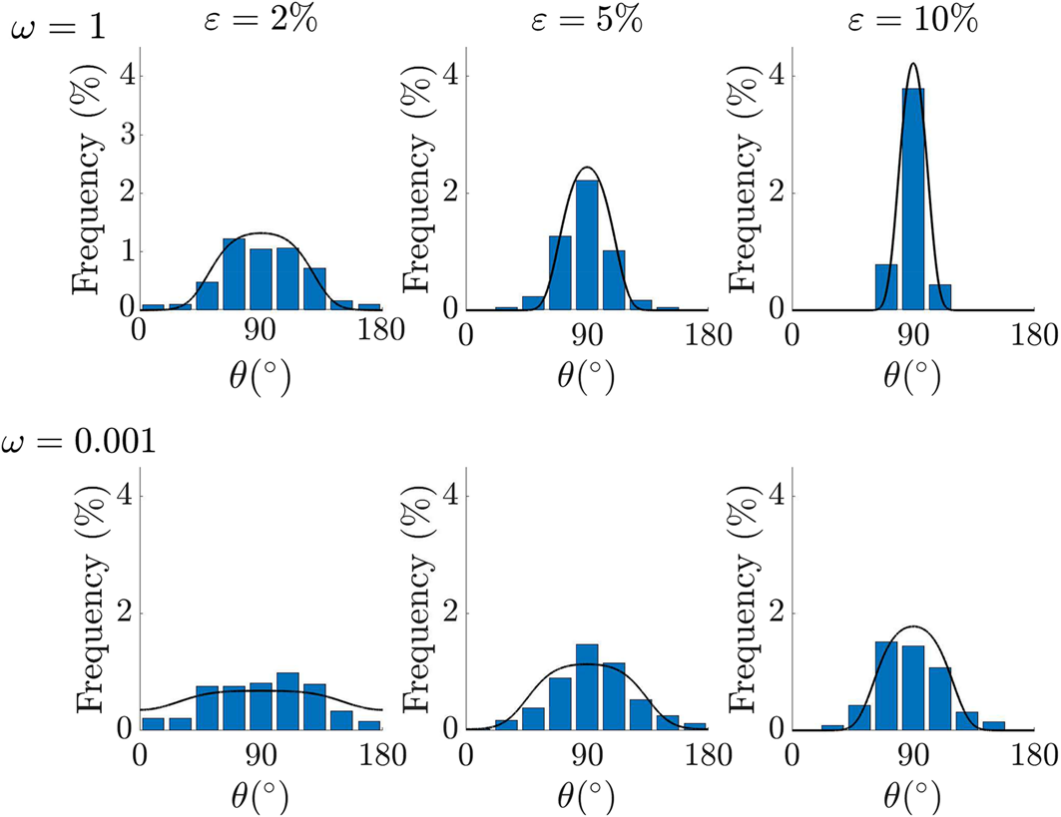
Equilibrium distributions (16) changing ${\bar{\sigma }}$, $\omega$ and $\varepsilon$, while $\sigma =0.2$ everywhere. The parameter $\varepsilon =2\%,5\%,10\%$ (first, second and third columns, respectively) is changed according to the experimental setup giving rise to decreasing values of ${\bar{\sigma }}^2=\dfrac{\sigma ^2}{2\varepsilon ^2}$ in the first row and ${\bar{\sigma }}^2=\dfrac{\sigma ^2}{2\varepsilon ^2\lambda \omega }$ with $\lambda =100$ s in the second row. The value of ${\tilde{K}}_s=0.7$ is used.

Focusing on the stationary distributions, Mao et al. (2021) report some experimental data in histograms over $[0^\circ , 180^\circ )$, changing the stretching amplitude (${\varepsilon }=2\%,5\%,10\%$) and frequency ($\omega =1$ Hz, 0.001 Hz). In particular, they show that increasing values of both amplitude and frequency lead to more peaked distributions. In their case, $r=0$ and the equilibrium orientation is perpendicular to the main stretching direction, i.e. $\theta _{eq}^1=90^\circ$. Trivially, due to symmetry, in this case the mode and the mean computed in $[0^\circ ,180^\circ )$ coincide, with $\sigma , \varepsilon$ and $\omega$ determining only the variance of the probability density function. In Fig. 6 in order to replicate the data reported by the histograms by Mao et al. 2021, we plot (16) where we set the same $\sigma =0.2$ and vary ${\varepsilon }$ and $\omega$. When $\omega =1$ (top row of Fig. 6), that corresponds to a high frequency regime, increasing the strain amplitude, coherently with the fact that $\bar{\sigma }^2=\dfrac{\sigma ^2}{2{\varepsilon }^2}$ (so, it goes like ${\varepsilon }^{-2}$) we have more peaked distributions that fit quite well the experimental distributions.

For $\omega =0.001 \text {Hz}$ since $\lambda \omega$ corresponds to a low frequency regime (it is $\lambda \omega =0.1$ if we take $\lambda =100$ s), we use $\bar{\sigma }^2=\dfrac{\sigma ^2}{2\varepsilon ^2\lambda \omega }$. Also in this case, the distributions peak up when increasing the strain amplitudes and, the theoretical results compare well with the experimental results, in spite of the fact that we are not really using a viscoelastic model but only taking into account of viscoelastic effects through a modification of ${\bar{\sigma }}$ that is valid in the low frequency regime. Comparing the results obtained for a fixed ${\varepsilon }$ at the different $\omega$’s (for instance, the last column in Fig. 6) simulations give more peaked distributions for higher frequencies.

**Fig. 7:**
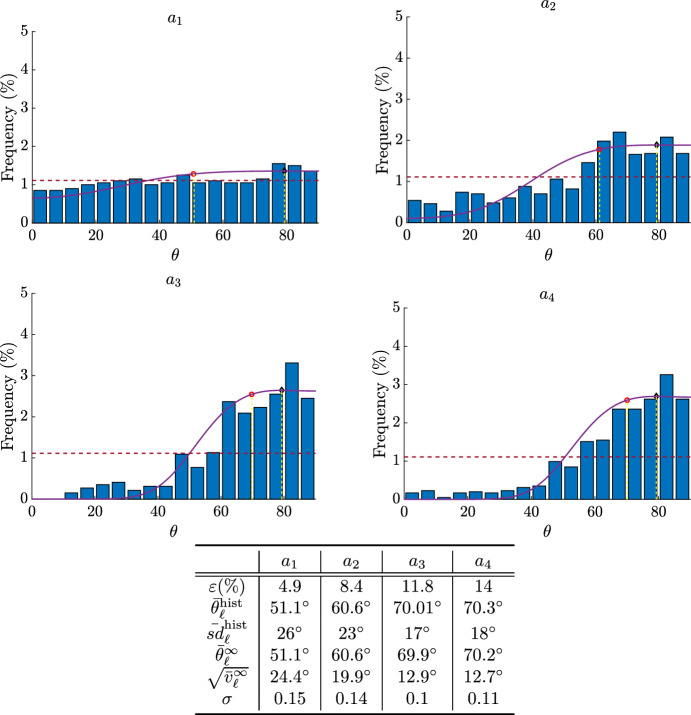
Equilibrium distributions (16) with $\bar{{\mathcal {U}}}$ given by (2) in the cases $a_1,a_2,a_3,a_4$ reported in the work by Faust et al. (2011) with applied strains listed in the table. In all figures we have $r=0.15$ and ${\tilde{K}}_s=0.7$ that allowed to best reproduce the averages of the histograms $\bar{\theta }_\ell ^{\text {hist}}$ by varying $\sigma$ in (16). The red circles represent the average circular orientation $\bar{\theta }_\ell ^{\infty }$ computed using (18). The black diamond represents $\theta _{eq}^1$. We also computed the standard deviation of the histogram ${\bar{sd}}_\ell ^{\text {hist}}$ and the standard deviation $\sqrt{{\bar{v}}_\ell ^{\infty }}$ of the stationary state using (20).

Faust et al. (2011) report the results of some experiment characterized by an evaluated biaxiality ratio of $r=0.15$. Assuming that $\alpha =\alpha _L$, as also suggested in Livne et al. (2014), the minimum elastic energy and therefore the mode is obtained at $\theta _{eq}^1\approx 79^\circ$. They perform the experiment applying different stretching amplitudes, namely $4.9\%$ (denoted as Case $a_1$), $8.4\%$ (Case $a_2$), $11.8\%$ (Case $a_3$), and $14\%$ (Case $a_4$). We recall that in this case, at variance with the (symmetric) one in Mao et al. (2021), the mean changes with the strain amplitude that influences ${\bar{\sigma }}$ (see second row in the table in Fig. 7). The means of the stationary distribution obtained by the simulation reported in the fourth row in the table closely follow the experimental ones. A slight difference is found for the standard deviation, expecially for larger amplitudes. Therefore, in Fig. 7 we compare their experimental results with the stationary probability density functions defined by (16) having average and standard deviation as computed from the histograms reported in Faust et al. (2011). In particular, we renormalize (16) over $[0,\pi /2)$ for comparison purposes with the histograms that are reported in the work by Faust et al. (2011). We remark again that, as the average is close to $\theta _{eq}$ in the presented cases, there may be two different values of $\sigma$ and therefore two different probability density functions that allow to recover the same average $\bar{\theta }_\ell$ (see Fig. 4). Here, we have chosen the one that allows to better reproduce the histograms.

## Kinetic Description

With the aim to get closer to the intrinsic dynamics followed by the single cell, in this section we will apply some classical tools of kinetic theory that, starting from the definition of the microscopic dynamics performed by the cells in the reorientation process, allow to derive the related mesoscopic evolution equation, such as (11). After going through the general procedure, we will then apply it to different microscopic rules. Then, in Sect. 4.2 we will discuss a different intrinsic dynamics that is probably performed by the cell, that through an optimal control argument drives them towards the most convenient orientation.

### Derivation of Kinetic Models from Discrete Random Processes

As a first step we formalize a microscopic discrete-in-time random process for describing the reorientation of cells. Let $\Theta _t\in [0,\pi )$ denote a random variable describing the orientation of a representative cell at time *t*. As typically done in kinetic theory (Pareschi and Toscani 2013), over a finite time interval $\Delta t$, we assume that a cell can change its main axis according to whether a reorientation occurs or not. We then express this discrete-in-time random process as

23 $$\begin{aligned} \Theta _{t + \Delta t} = (1-T_{\lambda _\theta })\Theta _{t} + T_{\lambda _\theta }\Theta _t' , \end{aligned}$$

where $\Theta _t'$ is the random variable in $[0,\pi )$ describing the new direction after a reorientation given the previous direction $\Theta _t$, while $T_{\lambda _\theta }$ is a Bernoulli random variable which we assume to be independent of all the other variables appearing in (23), discriminating whether the direction changes ($T_{\lambda _\theta }=1$) or not ($T_{\lambda _\theta }$=0) during the time interval $\Delta t$. In particular we set

24 $$\begin{aligned} Prob(T_{\lambda _\theta }=1)=\Delta t/\lambda _\theta , \end{aligned}$$

where naturally the necessary condition for $T_{\lambda _\theta }$ to be a random variable is $\Delta t\le \lambda _\theta$. Thus, the larger the time interval is, the higher the probability of having a reorientation is. The quantity $\Theta _t'$ models the change of direction (if it happens) and it may be generally expressed as

25 $$\begin{aligned} \Theta _t'=h_{\lambda ,K}(\Theta _t)+\sqrt{\sigma ^2}\xi \quad \text {mod}(\pi ), \end{aligned}$$

i.e., the new direction $\Theta _t'$ is a function $h_{\lambda ,K}$ of the previous orientation $\Theta _t$ and of the deformation parameters $\lambda _x,\lambda _y,K_{\Vert },K_{\bot }, K_s$, accounted for by the index $\lambda ,K$. We shall assume $h_{\lambda ,K}$ to be a regular function of its arguments, i.e. $h_{\lambda ,K} \in {\mathcal {C}}^1([0,\pi ))$, $\xi$ is a standard gaussian random variable, i.e. $\xi \sim {\mathcal {N}}(0,1)$ satisfying $\langle \xi \rangle =0$, $\langle \xi ^2\rangle =1$, while the term $\text {mod}(\pi )$ models the fact that $\Theta _t$ is $\pi$-periodic.

The aggregate description of the orientations of the cells can be obtained by determining the evolution of an observable quantity $\varphi =\varphi (\theta )$ defined on the phase space $[0,\pi )$. Taking into account the rules (23) together with the assumed independence of $T_{\lambda _\theta }$ it is possible to see that the evolution of the probability density function $f(t,\theta )$ is (see “Appendix A” for a formal derivation)

26 $$\begin{aligned} \begin{aligned} \frac{d}{dt}\int _0^{\pi }\varphi (\theta )f(t,\,\theta )\,d\theta = \dfrac{1}{\lambda _\theta }\langle \int _0^{\pi }\left( \varphi (\theta ')-\varphi (\theta ) \right) f(t,\, \theta ) d\theta \rangle , \end{aligned} \end{aligned}$$

where, coherently with (25), $\theta '$ is given by

27 $$\begin{aligned} \theta '=h_{\lambda ,K}(\theta )+ \sqrt{\sigma ^2}\xi \quad \text {mod}(\pi ). \end{aligned}$$

Equation (26) is a Boltzmann-type integro-differential equation. Choosing $\varphi (\theta )=1$ we readily obtain

$$\begin{aligned} \frac{d}{dt}\int _0^{\pi } f(t, \theta )\,d\theta =0, \end{aligned}$$

which means that the total mass of the agents is conserved in time by the interactions (27). Classically, the evolution of the statistical moments of *f* are obtained choosing $\varphi (\theta )=\theta ^n$, $n=0,\,1$, or circular moments may be recovered by setting $\varphi (\theta )=\cos (\theta ), \sin (\theta )$.

As shown in “Appendix B”, by an asymptotic procedure called *quasi-invariant limit* (see, for instance, Cordier et al. 2005; Furioli et al. 2017; Toscani 2006) based on a rescaled microscopic rule

28 $$\begin{aligned} \theta '=\theta +\gamma \left( h_{\lambda ,K}(\theta )-\theta \right) +\sqrt{\gamma {\sigma ^2}}\xi , \quad \text {mod}(\pi ), \end{aligned}$$

where $\gamma \ll 1$, we can obtain in the limit $\gamma \rightarrow 0$ a Fokker–Planck equation for the evolution of *f*

$$\begin{aligned} \dfrac{\partial }{\partial \tau }f(\tau ,\theta )=-\dfrac{1}{\lambda _\theta }\dfrac{\partial }{\partial \theta }\left[ (h_{\lambda ,K}(\theta ) -\theta )f(\tau ,\theta )\right] +\dfrac{1}{2\lambda _\theta }\dfrac{\partial ^2 }{\partial \theta ^2}\left[ \sigma ^2 f(\tau ,\theta )\right] . \end{aligned}$$

In particular, if we want to model the new orientation of a cell that tries to minimize a potential energy ${\mathcal {U}}$ after a time interval *dt* we may observe that the discrete in time random process describing the evolution of the orientation $\Theta _t$ happens with frequency $1/\lambda _\theta$ and may be expressed by discretizing (10) over *dt* (where we consider the high frequency regime) and setting $d t=\gamma$

29 $$\begin{aligned} \theta '=\theta -\gamma \varepsilon ^2\dfrac{\partial \bar{{\mathcal {U}}}}{\partial \theta }+\sqrt{\gamma \sigma ^2}\xi \quad \text {mod}(\pi ). \end{aligned}$$

Using the quasi-invariant limit procedure, we have the Fokker–Planck equation

$$\begin{aligned} \dfrac{\partial }{\partial \tau }f(\tau ,\theta )=\dfrac{\varepsilon ^2}{\lambda _\theta }\dfrac{\partial }{\partial \theta }\left( \dfrac{\partial \bar{{\mathcal {U}}}}{\partial \theta }f(\tau ,\theta )\right) +\dfrac{1}{2\lambda _\theta }\dfrac{\partial ^2 }{\partial \theta ^2}\left[ \sigma ^2 f(\tau ,\theta )\right] , \end{aligned}$$

which is, as expected, the same as (11).

### Reorientation as an Optimal Control Problem

In this section we want to introduce a new point of view consisting in modelling reorientation as a result of an internal control actuated by the cell that tries to minimize the elastic energy ${\mathcal {U}}$. From the mathematical point of view, this approach consists in expressing reorientation rules like (27) starting from a control problem, in the sense that we assume that the cell changes its orientation by a rotation angle $\nu \psi _{opt}$ where $\psi _{opt}$ is the angle that minimizes a certain cost functional ${\mathcal {J}}$. At the kinetic level, this has been widely treated in recent literature, e.g. by Preziosi et al. (2021), Tosin and Zanella (2019), Albi et al. (2020), Dimarco et al. (2022), Albi et al. (2014), Albi et al. (2014). Therefore, we write

30 $$\begin{aligned} \theta '=\theta +\nu \psi _{opt}, \quad \psi _{opt}=\text {argmin}_\psi {\mathcal {J}}(\psi ), \end{aligned}$$

where ${\mathcal {J}}$ is an energy functional defined as

$$\begin{aligned} {\mathcal {J}}(\psi )=\nu \dfrac{\psi ^2}{2}+\langle g(\theta ')\rangle , \end{aligned}$$

where the first contribution is a kinetic energy related to the control process, being $\nu$ a penalization coefficient, and the function *g* will be specialized later on.

In order to determine the optimal control at each reorientation, we need to introduce a Lagrangian

31 $$\begin{aligned} {\mathcal {L}}(\theta ',\psi )={\mathcal {J}}(\psi )+\chi \langle \theta '-(\theta +\nu \psi )\rangle , \end{aligned}$$

where $\chi \in {\mathbb {R}}$ is the Lagrange multiplier associated with the constraint (30). The optimality conditions are then identified by the solution of

32 $$\begin{aligned} \left\{ \begin{aligned} \dfrac{\partial {\mathcal {L}}}{\partial \theta '}&=\left\langle \dfrac{dg}{d\theta '}(\theta ')\right\rangle +\chi =0,\\ \dfrac{\partial {\mathcal {L}}}{\partial \psi }&=\nu (\psi -\chi )=0. \end{aligned} \right. \end{aligned}$$

Therefore, eliminating the Lagrange multiplier, the optimal value is implicitly identified by

33 $$\begin{aligned} \psi _{opt}+\left\langle \dfrac{dg}{d\theta '}(\theta ')\Big |_{\theta '=\theta +\nu \psi _{opt}}\right\rangle =0\,. \end{aligned}$$

If we choose $g={\varepsilon }^2\bar{{\mathcal {U}}}$, then Eq. (33) specializes to

$$\begin{aligned} \psi _{opt}+{\varepsilon }^2\dfrac{d\bar{{\mathcal {U}}}}{d\theta }(\theta +\nu \psi _{opt})=0, \end{aligned}$$

that, in general, allows to determine the optimal control only implicitly.

In any case the reorientation rule (30) specializes into

$$\begin{aligned} \theta '=\theta +\nu \psi _{opt}=\theta -\nu {\varepsilon }^2\dfrac{d\bar{{\mathcal {U}}}}{d\theta }(\theta +\nu \psi _{opt}), \end{aligned}$$

that in the limit of small $\nu$ used for the grazing limit and adding the stochastic term is equivalent to (29) and leads again to (12).

In order to explicitly determine the control, we can, instead, more classically take a quadratic form for *g*

34 $$\begin{aligned} g(\theta ')=\dfrac{{\varepsilon }^2}{2}[\theta '-{\hat{\theta }}(\theta )]^2\,, \end{aligned}$$

where, assuming to work in Case 1,

35 $$\begin{aligned} {\hat{\theta }}(\theta )=\theta _{eq}^1p(\theta )+(1-p(\theta ))(\pi -\theta _{eq}^1) \end{aligned}$$

with $p(\theta )$ a non negative and continuous function defined on $[0,\pi )$ that satisfies

36 $$\begin{aligned}{} & {} p(\theta _{eq}^1)=1 \quad p(\pi -\theta _{eq}^1)=0,\quad p(0)=p(\pi /2)=p(\pi )=1/2, \nonumber \\{} & {} p'(\theta _{eq}^1)=p'(\pi -\theta _{eq}^1)=0 \end{aligned}$$

in such a way that

37 $$\begin{aligned} {\hat{\theta }}(\theta _{eq}^1)=\theta _{eq}^1\qquad \textrm{and}\qquad {\hat{\theta }}(\theta _{eq}^2)=\theta _{eq}^2\,. \end{aligned}$$

Therefore, the choice of *g* given by (34)–(37) has the same extrema $\theta _{eq}^1$ and $\theta _{eq}^2$ as ${\bar{U}}$. The latter models, essentially, the fact that if $\theta$ is already close to an equilibrium orientation, then it is more likely not to change. In particular, we shall consider a second order polynomial satisfying the previous conditions. In this case one can explicitly solve (33) and determine

$$\begin{aligned} \psi _{opt}=-\,\dfrac{\varepsilon ^2}{1+\nu \varepsilon ^2}(\theta -{\hat{\theta }}), \end{aligned}$$

and therefore the reorientation rule (30) becomes

38 $$\begin{aligned} \theta '=\theta +\gamma {\varepsilon }^2({\hat{\theta }}(\theta )-\theta )\qquad \textrm{where}\quad \gamma =\dfrac{\nu }{1+\nu \varepsilon ^2}\,. \end{aligned}$$

Adding a stochastic fluctuation weighted by $\sigma _c$ we have

39 $$\begin{aligned} \theta '=\theta +\gamma {\varepsilon }^2({\hat{\theta }}(\theta )-\theta ) +\sqrt{\gamma \sigma _c^2}\xi \quad \text {mod} (\pi ). \end{aligned}$$

This rule implies the fact that at each reorientation the cell will activate a control to reach a better orientation that is given by a rotation of $\gamma {\varepsilon }^2({\hat{\theta }}(\theta )-\theta )$ (plus a white noise). This process will stop when the cell has oriented along the stable equilibria, because of the choice (34)–(37). In the symmetry points $\theta =0,\pi /2,\pi$ the cell has the same probability ($=1/2$) of reorienting either towards $\theta _{eq}^1$ or $\theta _{eq}^2=\pi -\theta _{eq}^1$.

As illustrated in Sect. 4.1, in this case the quasi-invariant direction limit procedure leads to the following Fokker–Planck equation

40 $$\begin{aligned} \dfrac{\partial }{\partial \tau }f(\tau ,\theta )= -\,\dfrac{{\varepsilon }^2}{\lambda _\theta }\dfrac{\partial }{\partial \theta }\left[ ({\hat{\theta }}(\theta )-\theta )f(\tau ,\theta )\right] +\dfrac{1}{2\lambda _\theta }\dfrac{\partial ^2}{\partial \theta ^2}\left[ \sigma _c^2 f(\tau ,\theta )\right] , \end{aligned}$$

that can be coupled with boundary conditions *F*3. Therefore, the stationary state is given by

$$\begin{aligned} - (\hat{\theta }(\theta )-\theta )f^\infty (\theta )+\dfrac{\partial }{\partial \theta }\left[ \bar{\sigma }_c^2f^\infty (\theta )\right] =0, \end{aligned}$$

where $\bar{\sigma }_c^2=\dfrac{\sigma _c^2}{2{\varepsilon }^2}$, that gives

41 $$\begin{aligned} f^{\infty }(\theta )=C\exp \left( \int _0^{\theta } \dfrac{{\hat{\theta }}(\theta )-\theta }{\bar{\sigma }_c^2} d\theta \right) \end{aligned}$$

where *C* is the normalization constant. This distribution has actually mode $\theta _{eq}^1$ and $\pi -\theta _{eq}^1$ in $[0,\pi )$, thanks to the choice (34)–(37) and average depending on the value of $\sigma _c$.

**Fig. 8:**
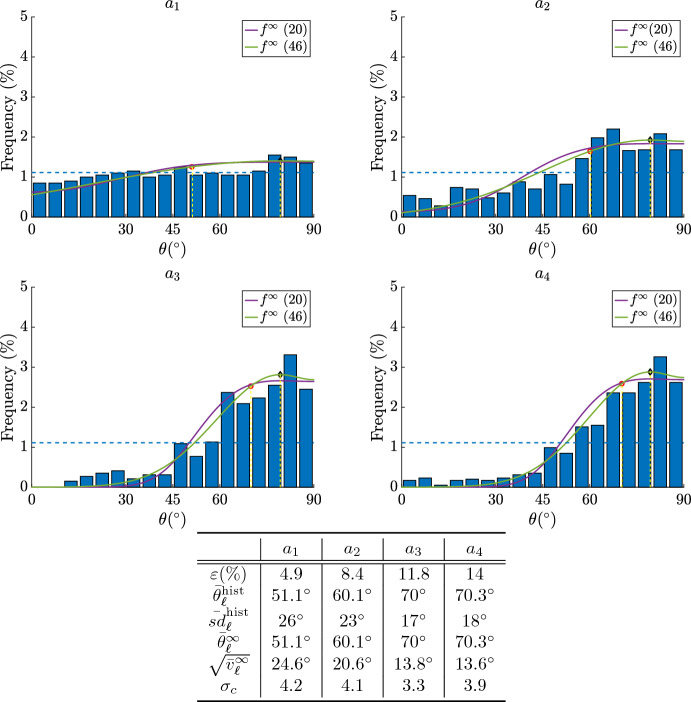
Equilibrium distributions (41) with $\bar{\sigma }_c^2=\dfrac{\sigma _c^2}{2{\varepsilon }^2}$ in the cases $a_1,a_2,a_3,a_4$ as listed in the table. As in Fig. 7, in all figures we have $r=0.15$ and ${\tilde{K}}_s=0.7$ that allowed to best reproduce the averages of the histograms $\bar{\theta }_l^{\text {hist}}$ by varying $\sigma _c$ in (41). The red circles represent the average circular orientation $\bar{\theta }_l^{\infty }$ computed using (18) with (41). The black diamonds represent $\theta _{eq}^1$. We also computed the standard deviation of the histogram ${\bar{sd}}_\ell ^{\text {hist}}$ and the standard deviation $\sqrt{{\bar{v}}_\ell ^{\infty }}$ of the stationary state using (20) with (41). We also superpose (16) with $\bar{{\mathcal {U}}}$ given by (2) as reported in Fig. 7

In Fig. 8 we compare the stationary distribution (41) with the experimental data by Faust et al. (2011), as in Fig. 7. Setting $\sigma _c$ in such a way that $\bar{\theta }_l$ of (41) is the same as in the work by Faust et al. (2011), we find that the microscopic rule (39) allows to recover probability density functions (41) that are better than those in Fig. 7. The prediction of the standard deviation, reported by the fourth line of the table in the two figures, shows that those of (41) are slightly closer to the linear standard deviation reported by Faust et al. (2011). We remark that the values of $\sigma$ and $\sigma _c$ are very different, and this is due to the fact that the rule (10) expresses the variation of $\theta$ in terms of its derivative and of the elastic energy, while (39) expresses the variation through a rotation angle that the cell performs during a reorientation.

**Fig. 9:**
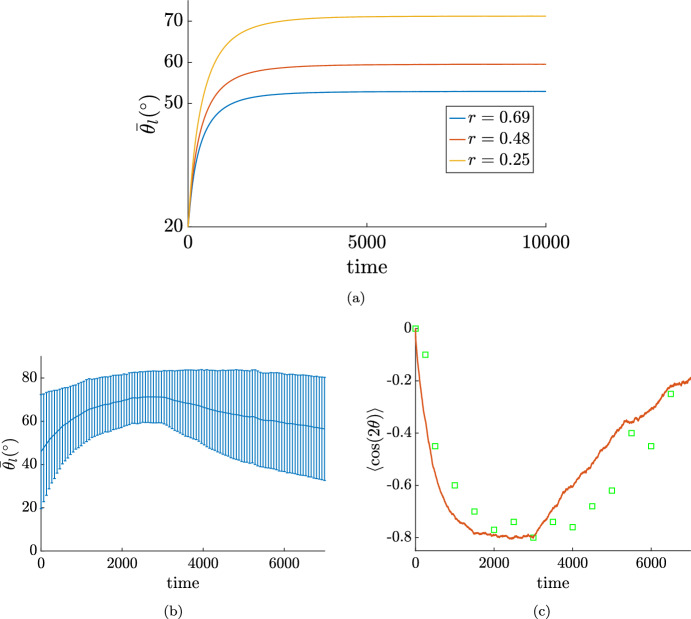
Temporal evolution of the mean of the orientation distribution. In (**a**) $\omega =1.2$ Hz and $\varepsilon =10\%$ as reported by Livne et al. (2014). In addition, $\lambda _{\theta }=6.6$ s and $\sigma _c=0.7$. In (**b**) and (**c**) $\omega =2$ Hz and $\varepsilon =8\%$ as reported in Jungbauer et al. (2008). In addition, $\lambda _{\theta }=6.6$ s and $\sigma _c=1.6$. After 3000 s stretching stops and cells tend to reorient uniformly. The standard deviation (one confidence interval) of the angle is also given in (**b**). In **c** the same mean is reported in terms of its $\cos 2\theta$ for a more direct comparison with the work by Jungbauer et al. 2008. Green squares correspond to the experimental results reported by Jungbauer et al. (2008).

Focusing on the temporal evolution of (40) in Fig. 9(a) we report the results obtained performing a Monte Carlo simulation of (23), (24), (39) with $N=10^6$ particles, $\gamma =10^{-2}$, as done in a different context for example by Loy and Tosin (2021). In fact, equation (40) is derived as the quasi-invariant limit of a Boltzmann like equation (see “Appendix B”) with microscopic rule (39), that is derived from (23)-(24)-(25) in the limit of large *N* and small $\Delta t$. In particular, we choose the data of the experimental results reported by Livne et al. (2014) where $\varepsilon =10\%$, $\lambda _{\theta }=6.6$ s and $\omega =1.2$ Hz, corresponding to a high frequency regime, and we set $\sigma _c=0.7$ so that the average orientation $\bar{\theta }_l$ of (41) with $\bar{\sigma }_c^2=\dfrac{\sigma _c^2}{2{\varepsilon }^2}$ is the same as reported in Livne et al. (2014). The qualitative behaviour corresponds to that reported in Livne et al. (2014). In particular we find that the rotation time is $\lambda _{\theta }/{\varepsilon }^2$ as stated in Livne et al. (2014).

Eventually, we want to replicate the experiment proposed by Jungbauer et al. (2008), where the authors stop stretching at a certain time and record the recovery phase towards a uniform distribution. To this aim, in Fig. 9b, c, we simulate (39) with $N=10^6$ elements, $\gamma =10^{-2}$ and using the parameters corresponding to the experiment reported in the work by Jungbauer et al. (2008): the stretch is imposed only for 3000 s, while ${\varepsilon }=8\%$ and $r=0.194$. After 3000 s, ${\varepsilon }=0$. We choose the same reorientation time as found in the work by Livne et al. (2014), i.e. $\lambda _{\theta }=6.6$ s, for the whole dynamics. Also in this case the behaviour corresponds to that reported in the work by Jungbauer et al. (2008) (green squares corresponds to the experimental results reported in Jungbauer et al. (2008)).

## Discussion

In order to describe the dynamics of cell reorientation under stretch, we have proposed a class of Fokker–Planck models for the evolution of the statistical distribution, i.e. the probability density function, paying particular attention to their link with the microscopic models. In particular, we have considered a stochastic microscopic process (10) in which the cell tends to minimize the elastic energy ${\mathcal {U}}$. The model is able to describe both the evolution and the stationary state of the probability density function over the orientations of the cells, which can be determined explicitly from as the stationary state of the Fokker–Planck equation relative to the SDE (10). The results compare well with several independent experiments (Faust et al. 2011; Hayakawa et al. 2001; Mao et al. 2021) showing the flexibility of the model.

In Sect. 4, we have used a well known procedure that allows to recover Fokker–Planck equations from microscopic stochastic discrete in time processes, through classical tools of kinetic theory. We have shown that by means of this approach it is possible to recover the Fokker–Planck equation (12) thanks to an appropriate choice of the microscopic rule for the evolution of the orientation angle. Then, thanks to the optimal control problem we have obtained a rule that is expressed as a function of the rotation angle performed by a cell during a reorientation. Also in this case the results compare well with several independent experiments (Faust et al. 2011; Jungbauer et al. 2008; Livne et al. 2014).

At present, the microscopic dynamics determining the drift term in the Fokker–Planck equation is defined according to biophysically sound qualitative arguments. In the future, the close link between the microscopic and the mesoscopic model shown here can be exploited, on the one hand, to better calibrate the model with respect to experimental data, and, on the other hand, to describe the microscopic mechanisms starting from measurements on the behaviour of single cells, whenever these data will be experimentally available. Moreover, the advantage of the microscopic rule (39) is that it is expressed in terms of rotation angles and is thus more amenable to possible extensions in order to include superposing effects that can be considered when dealing with cells seeded on a substratum, for example on collagen, that are subject to cyclic stretch, such as contact guidance and steric hindrance (Ristori et al. 2018). Moreover, the present framework may be extended to describe a three dimensional environment, by considering a second angle and its microscopic dynamic and a probability density function that depends on the two rotation angles.

## Appendix A

In this Appendix we want to show the formal derivation of (26) from the microscopic stochastic mechanism (23)–(25) as classically done, for example, in Pareschi and Toscani (2013). Let then $\varphi =\varphi (\theta )$ be an observable quantity defined on the phase space $[0,\pi )$. From (23) together with the assumed independence of $T_{\lambda _\theta }$, we see that the mean variation rate of $\varphi$ in the time interval $\Delta {t}$ satisfies

$$\begin{aligned} \frac{\langle \varphi \left( \Theta _{t+\Delta t}\right) \rangle -\langle \varphi \left( \Theta _{t}\right) \rangle }{\Delta {t}}= & {} \frac{\langle \varphi \left( (1-T_{\lambda _\theta })\Theta _{t} + T_{\lambda _\theta }\Theta _t'\right) \rangle -\langle \varphi \left( \Theta _{t}\right) \rangle }{\Delta {t}}\\= & {} \frac{\langle \varphi \left( \Theta _{t}\right) \rangle (1-\Delta t/\lambda _\theta ) + \langle \varphi \left( \Theta _t'\right) \rangle \Delta t/\lambda _\theta -\langle \varphi \left( \Theta _{t}\right) \rangle }{\Delta {t}}, \end{aligned}$$

where here and henceforth $\langle \Psi \rangle$ denotes the expectation of a generic random variable $\Psi$ with respect to its law. Then, the latter equality holds remembering that $\langle T_{\lambda _\theta }\rangle =\Delta t/\lambda _\theta$ and that $T_{\lambda _\theta }$ is independent from all the other random variables. Whence, we deduce the instantaneous time variation of the average of $\varphi$ in the limit $\Delta {t}\rightarrow 0^+$ as

$$\begin{aligned} \frac{d}{dt}\langle \varphi \left( \Theta _t\right) \rangle =\dfrac{1}{\lambda _\theta }\langle \big (\varphi \left( \Theta _t'\right) -\varphi \left( \Theta _t)\right) \rangle . \end{aligned}$$

If $f(t,\theta )$ is a probability density function, then we obtain

$$\begin{aligned} \begin{aligned} \frac{d}{dt}\int _0^{\pi }\varphi (\theta )f(t,\,\theta )\,d\theta = \dfrac{1}{\lambda _\theta }\langle \int _0^{\pi }\left( \varphi (\theta ')-\varphi (\theta ) \right) f(t,\, \theta ) d\theta \rangle , \end{aligned} \end{aligned}$$

where $\theta '$ is given by

$$\begin{aligned} \theta '=h_{\lambda ,K}(\theta )+ \sqrt{\sigma ^2}\xi \quad \text {mod}(\pi ). \end{aligned}$$

### Appendix B

One of the most relevant aspects of kinetic models is the possibility of characterising the stationary distributions arising asymptotically for $t\rightarrow +\infty$. This is typically carried out by means of asymptotic procedures, which, in suitable regimes of the parameters of the microscopic interactions, allows to transform a Boltzmann-type integro-differential equation into a partial differential equation, that is usually easier to be investigated analyticallly. A particularly efficient asymptotic procedure is the so-called *quasi-invariant limit*, which leads to *Fokker–Planck-type* equations.

The idea behind the quasi-invariant limit is that one studies a regime in which the new reorientation direction $\theta '$ is close enough to the previous direction $\theta$, so that the reorientation enhances a small variation. This concept was first introduced in the kinetic literature on multi-agent systems by Cordier et al. (2005), Toscani (2006) for binary collisions and by Furioli et al. (2017) for the interactions with a fixed background and has its roots in the concept of *grazing collisions* studied in the classical kinetic theory (Villani 1998).

In our framework this corresponds to considering a small reorientation and, then, a rescaled microscopic rule (27)

$$\begin{aligned} \theta '=\theta +\gamma \left( h_{\lambda ,K}(\theta )-\theta \right) +\sqrt{\gamma {\sigma ^2}}\xi , \quad \text {mod}(\pi ), \end{aligned}$$

where $\gamma \ll 1$. Now, diffusion is linked to a random variable $\sqrt{\gamma }\xi ={\mathcal {N}}(0,\gamma )$ with zero mean and variance $\gamma$. To compensate for the smallness of each reorientation, we simultaneously scale time as $\tau :=\gamma t$, which corresponds to observe the dynamics on a slower time scale and we introduce

$$\begin{aligned} f^{\gamma }(\tau ,\theta )=f(\tau /\gamma ,\theta ). \end{aligned}$$

Equivalently we can scale $\lambda _\theta$ as $\lambda _\theta ^\gamma :=\gamma \lambda _\theta$, meaning that the reorientation time corresponding to a small reorientation is shorter when $\gamma \ll 1$. Then, Eq. (26) rewrites

42 $$\begin{aligned} \frac{d}{d\tau }\int _0^{\pi }\varphi (\theta )f^{\gamma }(\tau ,\theta )\,d\theta =\dfrac{1}{\gamma \lambda _\theta } \langle \int _0^{\pi }\left( \varphi (\theta ')-\varphi (\theta ) \right) f^{\gamma }(\tau ,\theta ) d\theta \rangle , \end{aligned}$$

with (28). Now, let $\varphi \in {\mathcal {C}}^3([0,2\pi ))$ satisfy the requirement $\varphi (0)=\varphi (\pi )=0$, as we are considering quasi-invariant changes of a direction belonging to $[0,\pi )$ (Festa et al. 2018). Expanding the difference $\varphi (\theta ')-\varphi (\theta )$ in Taylor series about $\theta$ and using (28) we get

43 $$\begin{aligned} \begin{aligned} \frac{d}{d\tau }\int _0^{\pi }\varphi (\theta )f^{\gamma }(\tau ,\theta )\,d\theta&= \dfrac{1}{\gamma \lambda _\theta }\int _0^{\pi }\dfrac{d\varphi }{d\theta }(\theta )\gamma (h_{\lambda ,K}(\theta )-\theta )f^{\gamma }(\tau ,\theta ) \, d\theta \\&+\frac{1}{2\gamma \lambda _\theta }\int _0^{\pi }\dfrac{d^2\varphi }{d\theta ^2}(\theta )\gamma \sigma ^2 f^{\gamma }(\tau ,\theta )\,d\theta +R_\varphi (f^{\gamma })(\tau ,\theta ), \end{aligned} \end{aligned}$$

where

$$\begin{aligned} R_\varphi (f^{\gamma })(\tau ,\theta )=&\, \dfrac{1}{\gamma \lambda _\theta }\int _0^{\pi }\dfrac{1}{2}\gamma ^2 \dfrac{d^2\varphi }{d\theta ^2}(\theta ) (h_{\lambda ,K}(\theta )-\theta )\, d\theta \\&+\, \dfrac{1}{\gamma \lambda _\theta }\int _0^{\pi }\dfrac{1}{6} \dfrac{d^3\varphi }{d\theta ^3}(\theta +\delta (\theta '-\theta )) \langle (\theta '-\theta )^3\rangle \, d\theta , \end{aligned}$$

being $\delta \in (0,1)$. If we assume that $|\xi ^3|<\infty$ the following holds^1^ (cf. Cordier et al. (2005) for similar calculations)

44 $$\begin{aligned} \begin{aligned} \left|R_\varphi (f^{\gamma })(\tau ,\theta )\right|&\lesssim \left\| \dfrac{d^2\varphi }{d\theta ^2}\right\| _\infty \gamma \int _0^{\pi }(h_{\lambda ,K}(\theta )-\theta )^2f^{\gamma }(\tau ,\theta )\,d\theta \\&+\left\| \dfrac{d^3\varphi }{d\theta ^3}\right\| _\infty \int _0^{\pi }\left( \gamma ^2(h_{\lambda ,K}(\theta )-\theta )^3\right. \\&\left. +\sqrt{\gamma }4\sigma ^3 +3\gamma \sigma ^2(h_{\lambda ,K}(\theta )-\theta )^3\right) f^{\gamma }(\tau ,\theta )\,d\theta . \end{aligned} \end{aligned}$$

If we assume that $h^2_{\lambda ,K}, h^3_{\lambda ,K}$ are bounded in $[0,\pi )$, as *F*2 is satisfied, then

$$\begin{aligned} R_\varphi (f^\gamma )\xrightarrow {\gamma \rightarrow 0^+}0. \end{aligned}$$

Let us assume now that $(f^\gamma )$ converges in $C([0,\pi );\,L^1([0,\pi ))\cap L^1([0,\pi );\,\theta \,d\theta ))$, possibly up to subsequences, to a distribution function *f* when $\gamma \rightarrow 0^+$. Then, passing to the limit $\gamma \rightarrow 0^+$ in (43) we obtain the limit equation

$$\begin{aligned} \frac{d}{dt}\int _0^{\pi }\varphi (\theta )f(\tau ,\theta )\,d\theta&= \dfrac{1}{\lambda _\theta }\int _0^{\pi }\dfrac{d\varphi }{d\theta }(\theta )(h_{\lambda ,K}(\theta )-\theta )f(\tau ,\theta )\, d\theta \\&+\frac{1}{2\lambda _\theta }\int _0^{\pi }\dfrac{d^2\varphi }{d\theta ^2}(\theta )\sigma ^2f(\tau ,\theta )\,d\theta , \end{aligned}$$

which, by integration by parts, and recalling the compactness of the support of $\varphi$, can be recognised as a weak form of the following Fokker–Planck equation

$$\begin{aligned} \dfrac{\partial }{\partial \tau }f(\tau ,\theta )=-\dfrac{1}{\lambda _\theta }\dfrac{\partial }{\partial \theta }\left[ (h_{\lambda ,K}(\theta ) -\theta )f(\tau ,\theta )\right] +\dfrac{1}{2\lambda _\theta }\dfrac{\partial ^2 }{\partial \theta ^2}\left[ \sigma ^2 f(\tau ,\theta )\right] . \end{aligned}$$
